# Editorial: Immunity in the development of anti-cancer drug resistance

**DOI:** 10.3389/fphar.2022.1120037

**Published:** 2022-12-16

**Authors:** Ya Meng, Haitao Wang, Xuebing Li, Xinwei Wu, Heng Sun

**Affiliations:** ^1^ Guangdong Provincial Key Laboratory of Tumor Interventional Diagnosis and Treatment, Zhuhai People's Hospital, Zhuhai Hospital Affiliated With Jinan University, Zhuhai, China; ^2^ Thoracic Surgery Branch, Center for Cancer Research, National Cancer Institute (NCI), Bethesda, MD, United States; ^3^ Tianjin Key Laboratory of Lung Cancer Metastasis and Tumor Microenvironment, Tianjin Lung Cancer Institute, Department of Lung Cancer Surgery, Tianjin Medical University General Hospital, Tianjin, China; ^4^ Cancer Center, Faculty of Health Sciences, University of Macau, Macau, Macau SAR, China; ^5^ MOE Frontiers Science Center for Precision Oncology, University of Macau, Macau, Macau SAR, China; ^6^ Zhuhai Research Institute, University of Macau, Zhuhai, Macau SAR, China

**Keywords:** cancer therapy, drug resistance, immunity, precision oncology, tumor microenvironment

Development of resistance is the leading cause for cancer therapy failure (Vasan et al., 2019). Drug resistance could be innately generated before or during tumorigenesis; or acquired in response to cancer treatment. Multiple factors could affect the development of anti-cancer drug resistance, such as the mutation spectrum, cross-talks, and networks among pivotal signaling pathways of the cancer cells, as well as the tumor macro- and micro-environment, in which immunity is of the utmost importance (Wang et al., 2019). Actually, immunity itself serves as the first barrier against cancer initiation, and also works as the first “drug” to kill the cancer cells. In this Research Topic, we discussed the interplay between cancer and immunity, especially focusing on how cancer cells and immune cells affect each other during carcinogenesis and in the development of anti-cancer drug resistance.

It is well accepted that cancers are attributed to occurrence and accumulation of mutations, and the resulting abnormal activity and/or function of oncogenes and tumor suppressors. In addition to enhanced proliferation, survival, and anti-apoptosis capacities of cancer cells, the dysfunction of oncogenes and tumor suppressors is involved in the modulation of cancer immunity, which in turn protects cancer cells from immune surveillance and elimination. For example, Muthalagu et al. (2020) reported in pancreatic ductal adenocarcinoma (PDAC), activation of oncogenes Myc and KRAS could block the infiltration of NK cells *via* repressing type I interferon pathway, thus strengthen survival capacity to PDAC cells. In this Research Topic, Luo et al. reported that the KRAS-associated genes score correlated with the infiltration of several types of immune cells, including NK cells, memory CD4 T cells, plasma cells, and mast cells and might thus serve as a promising signature to distinguish the prognosis, molecular and immune characteristics of colon cancer patients. Besides, Hu et al. identified ST8SIA1, which plays an oncogenic role in triple-negative breast cancer (TNBC) (Nguyen et al., 2018), gliomas (Ohkawa et al., 2021), and other cancers, as a novel immune-related biomarker in clear-cell renal cell carcinoma (ccRCC). ST8SIA1 expression levels were negatively correlated with tumor purity and positively associated with infiltrated immune cells and expression of immune checkpoint genes. In addition, Xing et al. reviewed the recent findings of FBXW7, especially its tumor suppressive roles in multiple types of cancers, through regulating different immune cells for immune evasion and cancer development. Taken together, certain oncogenes and/or tumor suppressors not only play significant roles in initiating and fueling tumorigenesis, but also profoundly contribute to shaping the cancer immunity.

Aside from the influence of oncogenes and tumor suppressors, critical signaling pathways have been uncovered to control the drug sensitivities of cancer cells, such as the cancer cell dormancy regulated by Rb1-E2F signaling (Knudsen et al., 2019), epithelial to mesenchymal transition (EMT) induced by TGF-beta signaling (Katsuno et al., 2019), and so on. In this Research Topic, Zhu et al. investigated the roles of oxidative stress (OxS)-related genes in anti-cancer drugs sensitivity of lung adenocarcinoma (LUAD), and found a four OxS gene signature is correlated with the tumor mutation burden, tumor associated immune cell infiltration, and the expression of immune checkpoint molecules, which may contribute to individualized immunotherapeutic strategies for LUAD. Moreover, Feng et al. found that the peroxisome proliferator-activated receptor (PPAR) signaling pathway, related to fatty acid biosynthesis, might be a potential sorafenib resistance pathway in hepatocellular carcinoma (HCC) *via* regulating stemness of cancer cells. And in gastric cancer, Zhang et al. discovered that an EMT and immunity-related gene signature could be utilized as a biomarker to assess prognosis and guide precise treatment. Besides, epigenetic regulation such as DNA/RNA/histone modification, also broadly and deeply affects drug sensitivity. In this Research Topic, Meijing et al. identified three types of m6A methylation modification patterns are significantly different in immune infiltration in stomach adenocarcinoma (STAD). Further analysis by the researchers indicated that the m6A modification pattern may be a critical factor leading to inhibitory changes and heterogeneity in tumor micro-environment.

Anti-cancer drugs treatment not only targets the cancer cells but also reshapes the tumor macro-and micro-environment, which correspondingly affects the therapeutic efficacy of cancer patients. In this Research Topic, Tian et al. reviewed the critical roles of tumor micro-environment and immune escape in tumor occurrence, metastasis and anti-cancer drug resistance after sorafenib treatment in HCC patients. The relevant mechanisms focused on hypoxia, tumor-associated immune-suppressive cells, and immunosuppressive molecules. Moreover, Liu et al. found that macrophages and neutrophils are highly infiltrated, while CD8^+^ T cells are decreased in a sorafenib-resistant mouse HCC model. The authors identified nalidixic acid as a promising antagonist for sorafenib-resistant HCC treatment. In addition, Yang et al. investigated the roles of neutrophils in bladder cancer and established a neutrophil-based prognostic model incorporating five neutrophil-related genes, which may contribute to individualized prognostic prediction and clinical decision-making. Besides immune cells, inflammatory factors such as a series of cytokines and chemokines strongly modulate the responses of cancer cells to drugs. Wu et al. addressed the recent research progresses on regulating inflammatory factors for an intentional controlling anti-cancer response with immune checkpoint inhibitors. Indeed, the cross-talk between cancer cells and immune cells within the microenvironment, and the interplay between *in situ* cancers and the systematic immunity deserve more in-depth and detailed investigation in future, to further solve the mystery of anti-cancer drug resistance and shed light on the identification of novel and more effective drugs.

In recent years, cancer immunotherapies, including Chimeric Antigen Receptor (CAR) T cell and Immune Checkpoint Blockage (ICB) therapies, have achieved great success, though many knotty problems remain to be solved. Furthermore, with our deepening and broadening understanding of the roles and mechanisms of immunity in the development of anti-cancer drug resistance, more attention and effort will be paid to attacking the resistant cells from an immunomodulatory perspective in the near future, which we hope will eventually benefit cancer patients.

## References

[B1] Katsuno Y. Meyer D. S. Zhang Z. Shokat K. M. Akhurst R. J. Miyazono K. (2019). Chronic TGF-beta exposure drives stabilized EMT, tumor stemness, and cancer drug resistance with vulnerability to bitopic mTOR inhibition. Sci. Signal 12 (570), eaau8544. 10.1126/scisignal.aau8544 30808819 PMC6746178

[B2] Knudsen E. S. Pruitt S. C. Hershberger P. A. Witkiewicz A. K. Goodrich D. W. (2019). Cell cycle and beyond: Exploiting new RB1 controlled mechanisms for cancer therapy. Trends Cancer 5 (5), 308–324. 10.1016/j.trecan.2019.03.005 31174843 PMC6719339

[B3] Muthalagu N. Monteverde T. Raffo-Iraolagoitia X. Wiesheu R. Whyte D. Hedley A. (2020). Repression of the type I interferon pathway underlies MYC- and KRAS-dependent evasion of NK and B cells in pancreatic ductal adenocarcinoma. Cancer Discov. 10 (6), 872–887. 10.1158/2159-8290.CD-19-0620 32200350 PMC7611248

[B4] Nguyen K. Yan Y. Yuan B. Dasgupta A. Sun J. Mu H. (2018). ST8SIA1 regulates tumor growth and metastasis in TNBC by activating the FAK-AKT-mTOR signaling pathway. Mol. Cancer Ther. 17 (12), 2689–2701. 10.1158/1535-7163.MCT-18-0399 30237308 PMC6279518

[B5] Ohkawa Y. Zhang P. Momota H. Kato A. Hashimoto N. Ohmi Y. (2021). Lack of GD3 synthase (St8sia1) attenuates malignant properties of gliomas in genetically engineered mouse model. Cancer Sci. 112 (9), 3756–3768. 10.1111/cas.15032 34145699 PMC8409297

[B6] Vasan N. Baselga J. Hyman D. M. (2019). A view on drug resistance in cancer. Nature 575 (7782), 299–309. 10.1038/s41586-019-1730-1 31723286 PMC8008476

[B7] Wang X. Zhang H. Chen X. (2019). Drug resistance and combating drug resistance in cancer. Cancer Drug Resist 2 (2), 141–160. 10.20517/cdr.2019.10 34322663 PMC8315569

